# Perception of gender norms and its association with bullying behavior among adolescent students

**DOI:** 10.1186/s42506-024-00151-1

**Published:** 2024-03-11

**Authors:** Shaimaa S. Abdelrheem, Omaima El-Gibaly, Hasnaa Khairy

**Affiliations:** 1 Public Health and Community Medicine Department, Faculty of Medicine, Aswan University, Aswan, Egypt; 2 Armed Forces College of Medicine, Cairo, Egypt; 3 Public Health and Community Medicine Department, Faculty of Medicine, Assiut University, Assiut, Egypt

**Keywords:** Gender roles, Gender traits, Violence, Teenagers

## Abstract

**Background:**

Inequitable gender norms are increasingly seen as a risk to health and well-being. Although adolescence represents a critical phase of development before adulthood, there is limited understanding about adolescents’ perception of gender norms in Egypt. Adolescents’ nonconformity with stereotypical gender norms is suspected to increase their exposure to bullying behavior. This study aimed to explore the adolescents’ perception of gender norms — especially towards romantic relations and stereotypical gender traits and roles — and its association with bullying behavior among school children.

**Methods:**

A cross-sectional study of a sample of 400 students 11–17 years from 10 public schools in Aswan city, Egypt was conducted. Data were collected via interviewer-administered questionnaires using the Gender Norms Scales and School Life Survey tool for bullying.

**Results:**

School adolescents of both sexes showed no significant difference with respect to their attitudes towards romantic relationships engagement, but boys were slightly more permissive about romantic relationships than girls (54.4%, 46.1%, respectively). On the other hand, girls were significantly more likely to indicate agreement with a sexual double standard regarding boy/girl relationships than boys (75% vs. 46.4%, *P* < 0.001). Regarding gender stereotypical traits, girls’ and boys’ perception showed no significant difference, but boys had more endorsement, and 64.3% of boys perceived more agreement with gender stereotypical traits compared to 57.4% of girls. Also, boys were more likely to express more agreement with stereotypical gender roles than girls (74% vs. 52.9%, *P* < 0.001). However, 51% of students agreed that it was okay to tease a boy who acted like a girl, and 27.5% agreed that it was okay to tease a girl who acted like a boy. Students’ status of bullying and victimization was not significantly associated with any studied gender norms concept.

**Conclusions:**

Perception of unequal gender norms starts early in adolescence. Boys are more accepting of heteronormative relations among adolescents and more likely to endorse stereotypical gender roles than girls, while girls are more conservative and more likely to perceive a sexual double standard regarding boy/girl relationships. Moreover, adolescents of both genders perceived more agreement with deserving sanctions for atypical gender behaviors in boys with higher perception in boys compared to girls. However, both boys’ and girls’ perception of gender is not related to their status of bullying and victimization. This has important implications for understanding the development of gender norms and their impact on adolescent behavior and social interactions.

## Introduction

Adolescents (10–19 years) represent a large portion of the total population both globally (16%) and in Egypt (19%) [1, 2]. The adolescents’ successful transition to adulthood is shaped significantly by their societal context, including gender norms [3]. Transforming patriarchal norms and achieving gender equality are part of the fifth Sustainable Development Goals (SDGs) [4]. In light of that, in 2018, the Bellagio Working Group put adolescents at the center of strategies that increase gender equality [5].

Gender norms are defined as the widely accepted social rules about roles, traits, behaviors, status, and power associated with masculinity and femininity in a specific culture [6]. Inequitable gender norms, especially in low- and middle-income countries, lead to gender inequality which has many negative outcomes like gender-based discrimination in education and the workforce as well as gender-based practices such as early marriage and violence [7]. In Egypt, the Family Health Survey, 2021, shows that the percentage of women aged 20 to 24 years who were married before the age of 18 was 17.5%, while the percentage of those who married before the age of 15 was 0.9% [8].

Moreover, gender’s effect on health could be observed in multiple ways, including differential exposure to risk and differential access to healthcare and other material resources [9]. Hence, understanding adolescents’ perception of gender norms is an important step to build upon it. One of the other problems whose roots start in adolescence is violence. In Egypt, the Global School-based Student Health Survey (2012) found that 62% of Egyptian male adolescents were found to be involved in violent physical acts compared to only 29% of female adolescents. Furthermore, 70% of Egyptian adolescent students had been bullied [10], where bullying is a form of aggressive act that is repeated over time and involves an imbalance of power between the perpetrator and the victim [11].

Reviewing literature showed that gender norms might play a role in the extent to which adolescents experience bullying, as adolescents who do not conform to norms are more likely to be bullied. Additionally, adolescent girls may expect gender-based violence as part of the socialization process [12–14]. Our study aimed to explore the adolescents’ perception of gender norms and to investigate if there is an association between their perceptions of gender norms and their involvement in bullying behavior.

## Methods

### Study design, settings, and sample

A cross-sectional study was done in Aswan city, Egypt, from October 2019 to March 2020 and included 400 adolescent students. The students were chosen from 10 public schools out of 140 schools (primary, preparatory, and secondary) in the city using a multistage sampling method according to probability proportional to the schools’ population size. One class from each school was randomly picked as a cluster. The Epi Info software program version 7.1.5 was used to calculate the sample size. With a 95% confidence interval and a population size (number of students enrolled in primary, preparatory and secondary schools in Aswan city) of 93,790 students, the minimum sample size needed was 384 participants, assuming that 50% of the students had stereotypical gender norms and a 5% margin of error. The final total sample size was 404 students after adding 5% for possible nonresponse. Out of 443 students (10 groups with an average of 45 students) who were asked to join the study, 400 agreed to participate (a response rate of 90%).

### Data collection

#### Data collection tool

Data were collected using an interviewer-administered questionnaire which started with some sociodemographic data like age, sex, parents’ educational levels, parents’ occupation, and family structure (living with single or both parents). After that, the questionnaire was divided into two parts:

- *Gender norms*: Tools used in this study were part of the measures that were developed during phase 1 of the Global Early Adolescent Study (GEAS) [15]. The GEAS is the first international study to focus on gender norms and sexuality among early adolescents. The used measure of GEAS is the gender norms scale. It covered four dimensions: heteronormative romantic relationships among adolescents (six items), sexual double standards (four items), stereotypical gender traits (seven items), and stereotypical gender roles (five items). This instrument was formed by using themes identified in narrative interviews with adolescents and their parents to measure gender norms among young adolescents internationally. Items are all designed on a 5-point Likert scale (1: disagree a lot, 2: disagree a little, 3: neither agree nor disagree, 4: agree a little, 5: agree a lot), and it was tested for face validity. The internal reliability was assessed by computing polychoric ordinal Cronbach’s alpha [15]. Also, the Arabic version of the instrument was provided by Assiut University, Egypt, which is the Arabic partner in GAES project-phase I [15]. For the scoring procedure, scores were computed as a mean score across items of each subscale, with each individual score ranging from 1 to 5, based on the 5-point Likert scale [15, 16]. Then, by using the mean score as a cut-off point, the participants are classified to adolescents with less agreement and adolescents with more agreement of each studied gender norms concepts.
- *Bullying and victimization*: We used the School Life Survey tool to detect the frequency of bullying and victimization [17]. It is formed of a bullying scale and a victimization scale, and each of them contains many items which cover the three types of bullying and victimization (physical, verbal, and relational). It consists of 9 items for the bullying scale and 15 items for the victimization scale. We summed the items in each scale to give an overall score of bullying or victimization. The frequency of four acts during the past 4 weeks was the cut-off level for detecting bullying or victimization. Regarding the psychometric properties, Cronbach’s alpha of the whole scale is 0.83, and the 1-week test-retest reliability of the bullying scale and victimization scale was respectively 0.84 and 0.94 [17].

#### Data collection procedures

After taking the required administrative approvals from the local education directorates, we performed a pilot study on 30 students from different schools in Aswan city to test the questionnaire appropriateness and to estimate the time required to complete it. The pilot study cases were not included in the sample of the main study. After orienting each school principal, consent forms were sent to parents of the chosen students explaining the purpose and procedures of the study. After obtaining parents written consents and adolescent assents from students, we invited each student to a personal interview to complete the questionnaire through a 20- to 30-min period during lunch breaks or other school periods.

### Statistical analysis

Pre-coded data were entered on the computer using the Statistical Package for Social Science (SPSS) version 23. The data were summarized using mean and standard deviation for quantitative variables, while frequency and percentages were used for qualitative variables. Statistical differences between groups were tested using the *χ*^2^ test or its alternatives (Fisher’s exact, Monte Carlo simulation) for qualitative variables and the Mann-Whitney test and Kruskal Wallis test for quantitative ones. Moreover, binary logistic regression analysis was used to assess the effect of participants’ perception of the studied gender norms on bullying perpetration and victimization exposure. Significance was considered at a *P*-value ≤ 0.05.

## Results

A total of 400 school adolescents aged 11–16 years with a mean of 12.77 ± 1.68 years were enrolled for this study. The entire sample included 196 boys (49.0%) and 204 girls (51.0%). The percentage of students in primary and preparatory schools was almost equal, with 40.5% and 40.2%, respectively. The parental characteristics showed that a higher percentage of fathers (38.5%) had completed their university education compared to mothers (31.5%). Regarding their occupational status, only 3% of the students’ fathers did not work, while nearly 70% of the students’ mothers were housewives. The families of 91.8% of students were formed of married parents, while few of the students (8.2%) were living in single-parent households due to divorce, separation, or widowhood (Table 1). Table 1 also shows the adolescent students’ status with regard to bullying and victimization, where 30.2% of them were classified as victims and 29.8% of them were bully victims, while unique bullies (i.e., perpetrators) represented only 7.5% of the participated students.

**Table 1:** Demographic characteristics of the study sample of adolescent students in Aswan city, Egypt, 2019–2020

Variable	Frequency ***N*** = 400	Percent (%)
**Gender of the student**		
Male	196	49.0
Female	204	51.0
**Age(years)**		
Mean ± SD	12.77 ± 1.681	
**Educational level**		
Primary	162	40.5
Preparatory	161	40.2
Secondary	77	19.3
**Educational level of the father**		
Illiterate/literacy classes	41	10.2
Basic education [primary or preparatory]	28	7.0
Secondary/technical	177	44.2
University degree/higher	154	38.6
**Educational level of the mother**		
Illiterate/literacy classes	45	11.2
Basic education [primary or preparatory]	36	9.0
Secondary/technical	193	48.2
University degree/higher	126	31.6
**Working status of the father**		
Working for cash	388	97.0
Not working for cash/unemployed	12	3.0
**Working status of the mother**		
Working for cash	118	29.5
Not working for cash/ housewife	282	70.5
**Family structure**		
Living with two parents	367	91.8
Other^a^	33	8.2
**Bullying/victimization status**		
Bully	30	7.5
Victim	121	30.2
Bully victim	119	29.8
Neither bully nor victim	130	32.5

^a^Single parent or other caregivers

Table 2 shows the responses of boys and girls to the different statements related to the studied gender norms. There are significant differences between boys and girls for some of the statements, such as “It’s normal for a boy your age to want a girlfriend,” “Adolescent boys fool girls into having sex,” and “A man should have the final word about decisions in the home.”

**Table 2:** Gender norms among the study sample of adolescent students in Aswan city, Egypt, 2019–2020

Gender norms concept	Gender	Responses ***N*** (%)					***p***-value
		Disagree a lot	Disagree a little	Neither agree nor disagree	Agree a little	Agree a lot	
**Adolescent acceptance of romantic relationships**							
It is normal for a boy your age to want a girlfriend	Boys	33 (16.8)	10 (5.1)	8 (4.1)	52 (26.5)	93 (47.5)	0.044*
	Girls	21 (10.3)	6 (2.9)	17 (8.3)	71 (34.8)	89 (43.7)	
It is normal for a girl to want a boyfriend at your age	Boys	26 (13.3)	15 (7.7)	23 (11.7)	63 (32.1)	69 (35.2)	0.004*
	Girls	45 (22.1)	15 (7.4)	6 (2.9)	61 (29.9)	77 (37.7)	
A girl should be able to have a boyfriend if she wants	Boys	87 (44.3)	26 (13.3)	5 (2.6)	41 (20.9)	37 (18.9)	0.456
	Girls	107 (52.5)	27 (13.1)	3 (1.5)	32 (15.7)	35 (17.2)	
A boy should be able to have a girlfriend if he wants	Boys	60 (30.6)	24 (12.2)	5 (2.6)	34 (17.3)	73 (37.3)	0.001*
	Girls	78 (38.3)	37 (18.1)	7 (3.4)	45 (22.1)	37 (18.1)	
**Mean score** (1–5, 5 indicating strongest approval of adolescent romantic relationships) (mean +/− SD)	Boys	3.315 ± 1.071					0.056
	Girls	3.120 ± 1.044					
**Sexual double standard**							
Adolescent boys fool girls into having sex	Boys	32 (16.3)	29 (14.8)	8 (4.1)	57 (29.1)	70 (35.7)	< 0.001*
	Girls	5 (2.5)	19 (9.3)	4 (2.0)	51 (25.0)	125 (61.2)	
Adolescent girls should avoid boys because they trick them into having sex	Boys	9 (4.6)	18 (9.2)	5 (2.5)	44 (22.4)	120 (61.3)	< 0.001*
	Girls	5 (2.5)	21 (10.3)	0 (0)	17 (8.3)	161 (78.9)	
Girls are the victims of rumors if they have boyfriends	Boys	17 (8.7)	23 (11.7)	3 (1.5)	35 (17.9)	118 (60.2)	< 0.001*
	Girls	2 (1.0)	10 (4.9)	1 (0.5)	31 (15.2)	160 (78.4)	
Boys have girlfriends to show off to their friends	Boys	33 (16.8)	24 (12.3)	5 (2.6)	52 (26.5)	82 (41.8)	**0.001**
	Girls	12 (5.9)	17 (8.3)	10 (4.9)	51 (25.0)	114 (55.9)	
Adolescent boys lose interest in a girl after they have sex with her	Boys	13 (6.6)	15 (7.7)	13 (6.6)	48 (24.5)	107 (54.6)	0.021*
	Girls	5 (2.5)	7 (3.4)	20 (9.8)	39 (19.1)	133 (65.2)	
Boys tell girls they love them when they do not	Boys	28 (14.3)	34 (17.3)	12 (6.1)	54 (27.6)	68 (34.7)	< 0.001*
	Girls	9 (4.4)	22 (10.8)	12 (5.9)	53 (26.0)	108 (52.9)	
**Mean score** (1−5, 5 indicating strongest endorsement of sexual double standard) (mean +/− SD**)**	Boys	3.861 ± 0.832					< 0.001*
	Girls	4.366 ± 0.620					
**Stereotypical gender traits: toughness versus vulnerability**							
Boys should always defend themselves even if it means fighting	Boys	4 (2.0)	10 (5.1)	2 (1.0)	25 (12.8)	155 (79.1)	**< 0.001**+*
	Girls	6 (2.9)	25 (12.3)	1 (0.5)	58 (28.4)	114 (55.9)	
It is important for boys to show they are tough even if they are nervous inside	Boys	14 (7.2)	30 (15.3)	3 (1.5)	39 (19.9)	110 (56.1)	0.714
	Girls	17 (8.3)	38 (18.6)	4 (2.0)	45 (22.1)	100 (49.0)	
Boys who behave like girls are considered weak	Boys	16 (8.2)	33 (16.8)	4 (2.1)	32 (16.3)	111 (56.6)	0.57
	Girls	16 (7.8)	23 (11.3)	6 (2.9)	34 (16.7)	125 (61.3)	
Boys should be raised to be tough so can overcome any difficulties in life	Boys	13 (6.6)	20 (10.2)	2 (1.0)	25 (12.8)	136 (69.4)	0.2
	Girls	4 (2.0)	21 (10.3)	1 (0.5)	25 (12.2)	153 (75.0)	
Girls are expected to be humble	Boys	7 (3.6)	17 (8.7)	2 (1.0)	31 (15.8)	139 (70.9)	0.157
	Girls	15 (7.3)	28 (13.7)	3 (1.5)	34 (16.7)	124 (60.8)	
Girls should avoid raising their voice to be lady like	Boys	0 (0.0)	4 (2.0)	0 (0.0)	9 (4.6)	183 (93.4)	**0.034**+*
	Girls	4 (2.0)	4 (2.0)	2 (1.0)	19 (9.2)	175 (85.8)	
Girls need their parents’ protection more than boys.	Boys	6 (3.1)	12 (6.1)	1 (0.5)	12 (6.1)	165 (84.2)	0.856
	Girls	6 (2.9)	8 (3.9)	2 (1.0)	12 (5.9)	176 (86.3)	
**Mean score** (1–5, 5 indicating strongest endorsement of stereotypical gender traits) (mean +/− SD**)**	Boys	4.403 ± 0.527					0.076
	Girls	4.315 ± 0.548					
**Stereotypical gender roles**							
A woman’s role is taking care of her home and family	Boys	0 (0)	4 (2.0)	1 (0.5)	11 (5.6)	180 (91.9)	0.015+*
	Girls	5 (2.5)	14 (6.9)	2 (1.0)	11 (5.4)	172 (84.2)	
Boys and girls should be equally responsible for household chores	Boys	63 (32.1)	44 (22.4)	-	32 (16.3)	57 (29.2)	0.045*
	Girls	67 (32.8)	34 (16.7)	-	21 (10.3)	82 (40.2)	
A man should have the final word about decisions in the home	Boys	5 (2.6)	12 (6.1)	2 (1.0)	25 (12.8)	152 (77.5)	< 0.001*
	Girls	12 (5.9)	33 (16.2)	2 (1.0)	41 (20.1)	116 (56.8)	
A woman should obey her husband in all matters	Boys	4 (2.0)	36 (18.4)	3 (1.5)	53 (27.1)	100 (51.0)	0.105
	Girls	13 (6.4)	48 (23.5)	5 (2.5)	46 (22.5)	92 (45.1)	
Men should be the ones who bring money home for the family, not women	Boys	11 (5.6)	21 (10.7)	2 (1.0)	20 (10.2)	142 (72.5)	< 0.001*
	Girls	15 (7.4)	53 (26.0)	3 (1.5)	30 (14.6)	103 (50.5)	
**Mean score** (1–5, 5 indicating strongest endorsement of stereotypical gender roles) (mean +/− SD**)**	Boys	4.191 ± 0.676					< 0.001*
	Girls	3.822 ± 0.882					
**Sanctions for challenging gender roles**							
It is okay to tease a girl who acts like a boy	Boys	100 (51.1)	34 (17.3)	4 (2.0)	25 (12.8)	33 (16.8)	0.795
	Girls	105 (51.5)	40 (19.6)	7 (3.4)	21 (10.3)	31 (15.2)	
It is okay to tease a boy who acts like a girl	Boys	66 (33.7)	23 (11.7)	1 (0.5)	28 (14.3)	78 (39.8)	0.013*
	Girls	55 (27.0)	41 (20.1)	8 (3.9)	33 (16.2)	67 (32.8)	

+Monte Carlo simulation. *Significant

**Fig. 1:**
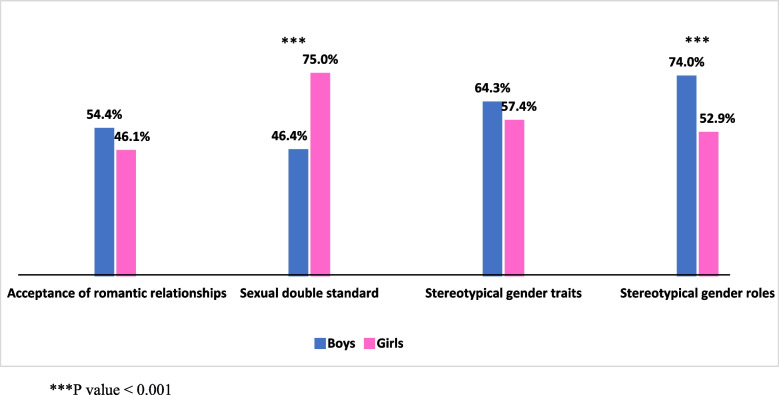
Percentages of the study participants with more agreement with gender norms by gender. ***P-value < 0.001

Our results found that there are significant differences between the perception of boys and girls for sexual double standards and stereotypical gender roles as 75% of girls had more agreement with questions related to sexual double standards compared to 46.4% of boys (*P* < 0.001), while 74% of boys had more agreement with stereotypical gender roles compared to 52.9% of girls (*P* = < 0.001). On the other hand, there are no significant differences between boys and girls for acceptance of romantic relationships and stereotypical gender traits. However, boys were more likely to indicate more agreement with adolescents’ romantic relationships than girls, with 54.4% and 46.1%, respectively (Fig. 1). Regarding sanctions for challenging gender roles, teasing boys who acted like girls was more accepted among the study participants than teasing girls who acted like boys (Table 2).

Searching for the association between the perception of gender norms and other sociodemographic characteristics (Table 3), the adolescents’ age was found to significantly affect only the perception of stereotypical gender roles as the participants’ mean age with more agreement with stereotypical gender roles was 12.6 years compared to 13.1 years for participants with less agreement (*P* = 0.021). Also, we found that there is a significant association between the students’ perception of stereotypical gender roles and the students’ educational level (*P* < 0.001). Adolescents who are in secondary level (54.5%) have less agreement with stereotypical gender roles than those who are in primary or preparatory levels (30.2%, 34.8%, respectively). In terms of the parents’ educational level and occupation, sexual double standards and stereotypical gender roles were found to be associated with the fathers’ educational level and mothers’ working status, while the mothers’ educational level was found to be associated with stereotypical gender traits and stereotypical gender roles. Students showed more agreement with these gender norms when their parents had lower levels of education and their mothers were employed. Lastly, we did not find a significant association between family structure and the studied gender norms.

**Table 3:** Gender norms among the study sample of adolescent students by some sociodemographic factors

Variable	Acceptance of romantic relationships		Sexual double standard		Stereotypical gender traits		Stereotypical gender roles	
	Less agreement (< the peer mean score) *N* (%)	More agreement (≥ the peer mean score)	Less agreement (< the peer mean score) *N* (%)	More agreement (≥ the peer mean score) *N* (%)	Less agreement (< the peer mean score) *N* (%)	More agreement (≥ the peer mean score) *N* (%)	Less agreement (< the peer mean score) *N* (%)	More agreement (≥ the peer mean score) *N* (%)
**Age**								
Mean +/− SD	**12.67** ± 1.67	**12.87** ± 1.68	**12.58** ± 1.65	**12.89** ± 1.69	**12.88** ± 1.72	**12.70** ± 1.65	**13.05** ± 1.77	**12.60** ± 1.60
***P*-value**	0.148		0.065		0.395		0.021*	
**Educational level**								
Primary	89 (54.9)	73 (45.1)	69 (42.6)	93 (57.4)	64 (39.5)	98 (60.5)	49 (30.2)	113 (69.8)
Preparatory	71 (44.1)	90 (55.9)	64 (39.8)	97 (60.2)	55 (34.2)	106 (65.8)	56 (34.8)	105 (65.2)
Secondary	39 (50.6)	38 (49.4)	23 (29.9)	54 (70.1)	38 (49.4)	39 (50.6)	42 (54.5)	35 (45.5)
***P*-value**	0.148		0.164		0.080		**0.001**	
**Educational level of the father**								
Illiterate/literacy classes	24 (58.5)	17 (41.5)	11 (26.8)	30 (73.2)	14 (34.1)	27 (65.9)	5 (12.2)	36 (87.8)
Basic education	12 (42.9)	16 (57.1)	13 (46.4)	15 (53.6)	9 (32.1)	19 (67.9)	15 (53.6)	13 (46.4)
Secondary/technical	85 (48.0)	92 (52.0)	54 (30.5)	123 (69.5)	67 (37.9)	110 (62.1)	57 (32.2)	120 (67.8)
University degree/higher	78 (50.6)	76 (49.4)	78 (50.6)	76 (49.4)	67 (43.5)	87 (56.5)	70 (45.5)	84 (54.5)
***P*-value**	0.056		0.001*		0.502		< 0.001*	
**Educational level of the mother**								
Illiterate/literacy classes	24 (53.3)	21 (46.7)	14 (31.1)	31 (68.9)	9 (20.0)	36 (80.0)	6 (13.3)	39 (86.7)
Basic education	17 (47.2)	19 (52.8)	11 (30.6)	25 (69.4)	17 (47.2)	19 (52.8)	12 (33.3)	24 (66.7)
Secondary/technical	91 (47.2)	102 (52.8)	73 (37.8)	120 (62.2)	69 (35.8)	124 (64.2)	61 (31.6)	132 (68.4)
University degree/higher	67 (53.2)	59 (46.8)	58 (46.0)	68 (54.0)	62 (49.2)	64 (50.8)	68 (54.0)	58 (46.0)
***P*-value**	0.967		0.173		0.003*		< 0.001*	
**Working status of the father**								
Working for cash	193 (49.7)	195 (50.3)	151 (38.9)	237 (61.1)	152 (39.2)	236 (60.8)	141 (36.3)	247 (63.7)
Not working for cash/unemployed	6 (50.0)	6 (50.0)	5 (41.7)	7 (58.3)	5 (41.7)	7 (58.3)	6 (50.0)	6 (50.0)
***P*-value**	0.986		1.000^#^		1.000^#^		0.370#	
**Working status of the mother**								
Working for cash	60 (50.8)	58 (49.2)	56 (47.5)	62 (52.5)	53 (44.9)	65 (55.1)	60 (50.8)	58 (49.2)
Not working for cash/housewife	139 (49.3)	143 (50.7)	100 (35.5)	182 (64.5)	104 (36.9)	178 (63.1)	87 (30.9)	195 (69.1)
***P*-value**	0.776		0.02*		0.133		< 0.001*	
**Family structure**								
Living with two parents	183 (49.9)	184 (50.1)	143 (39.0)	224 (61.0)	147 (40.1)	220 (59.9)	138 (37.6)	229 (62.4)
Other^**¤**^	16 (48.5)	17 (51.5)	13 (34.4)	20 (60.6)	10 (30.3)	23 (60.7)	9 (27.3)	24 (72.7)
***P*-value**	0.879		0.961		0.238		0.238	

#Fisher’s exact test. *Significant. ^**¤**^Single parent or another caregiver

Regarding the association between student’s perception of gender norms and bullying phenomena, we found that the students’ perception regarding heteronormative relationships, sexual double standard, stereotypical gender traits, and stereotypical gender roles has no significant association with bullying perpetration or victimization (Tables 4, 5).

**Table 4:** Binary logistic regression for gender norm subscales and bullying perpetration among the study participants

Model Variables®	Β	OR (95% ***CI***)	***p***-value
**Adolescent acceptance of romantic relationships (more agreement)**	0.312	1.36 (0.82–2.37)	0.341
**Sexual double standard (more agreement)**	0.125	1.13 (0.69–1.84)	0.614
**Stereotypical gender traits (more agreement)**	0.047	1.05 (0.65–1.69)	0.848
**Stereotypical gender roles (more agreement)**	0.291	1.34 (0.80–2.23)	0.266
**Constant**	2.345	10.438	0.415

Independent variable is bullying status (0 not bully, 1 bully)

®All the studied sociodemographic variables (age, gender, etc.) have been controlled in the model

Cox & Snell R2 = 0.116, Nagelkerke R2 = 0.159

**Table 5:** Binary logistic regression for gender norm subscales and victimization among the study participants

Model Variables®	Β	OR (95% ***CI***)	***p***-value
**Adolescent acceptance of romantic relationships (more agreement)**	0.301	1.35 (0.87–2.29)	0.381
**Sexual double standard (more agreement)**	0.139	1.15 (0.71–1.85)	0.568
**Stereotypical gender traits (more agreement)**	–0.347	0.71 (0.44–1.13)	0.144
**Stereotypical gender roles (more agreement)**	–0.256	0.77 (0.47–1.27)	0.310
**Constant**	2.174	8.79	0.442

Independent variable is victimization status (0 not victim, 1 victim)

®All the studied sociodemographic variables (age, gender, etc.) have been controlled in the model

Cox & Snell R2 = 0.084, Nagelkerke R2 = 0.113

## Discussion

The global development community has understood the importance of addressing the social and health needs of young people. It has also acknowledged the negative impact of inequitable gender norms on young people of both sexes. Therefore, it is important to measure the various dimensions of gender norms, especially among early adolescents who should be prioritized in strategies aimed at enhancing gender equality [5].

Our study used gender norms scales, which are part of the GEAS measures that were developed to be cross-cultural measures, so it could be used to monitor and compare gender norms across time and space and help in following global progress towards achieving the United Nations’ 5th Sustainable Development Goal on gender equality by 2030 [18].

These gender norm scales covered different dimensions: heteronormative romantic relationships, sexual double standard, and endorsement of stereotypical gender traits and roles.

Regarding acceptance of heteronormative romantic relationships among the participated adolescents, students of both sexes showed no difference in the acceptance of romantic relationships, but boys were slightly more permissive about romantic relationships than girls. This matches the results from the GEAS in Indonesia and Shanghai, where boys were found to be more likely to approve romantic relations during adolescence [19, 20]. Also, our findings agree with Vu et al. study in Uganda, which tested an adapted GEM scale on young adolescents, and came to the conclusion that boys exhibit more equitable attitudes about relationships than girls in adolescence [21].

Moreau et al. study suggested that the sexual double standard, which is suggestive of perceptions of unequal social status related to romantic relationships for boys and girls, starts early in adolescence and is found across different cultures [15]. More specifically Moreau and her colleagues found that girls scored higher than boys on the sexual double standard scale both in Assiut, Egypt, and Ghent, Belgium [15]. This pattern was also observed in Kinshasa, Democratic Republic of the Congo [22], where girls were more likely to indicate agreement with a sexual double standard regarding boy/girl relationships. This consistent pattern means that girls have more conservative views about relationships than boys, as they agree more with the sexual double standard subscale and are less accepting romantic relationships during adolescence. Therefore, more research is required to explore the different views boys and girls have regarding their expectations from romantic relationships in adolescence and to examine whether girls’ more cautious view is related to them experiencing unequal relationships or to generally show the two genders socialize.

Concerning stereotypical gender traits, our findings suggested that more than half of the boys and girls who participated in the study expressed more agreement towards stereotypical gender traits regarding male toughness and female vulnerability, and that boys were more likely to endorse stereotypical gender traits than girls; however, there was no significant difference between the genders. On the one hand, this is supported by the finding of the GEAS in Indonesia [19], which indicated that more than half of the adolescents agreed with the statements on stereotypical gender traits, except for “boys should always defend themselves even if it means fighting” (48% agreed) and “boys who behave like girls are considered weak” (43% agreed), and boys, generally, showed higher endorsement on norms that indicate boys’ toughness over girls’ vulnerabilities. This was observed in other localities such as the following: Flanders, Belgium and Cuenca, and Ecuador [23]. On the other hand, most adolescents in Shanghai rejected stereotypical gender traits, but boys were also more likely to endorse stereotypical gender traits than girls [20].

The present study findings of stereotypical gender roles regarding household decisions and division of labor and responsibilities showed that boys were more likely to support stereotypical gender roles than girls. Similarly, more than half of early adolescents in Indonesia supported stereotypical gender roles, but boys showed higher endorsement of males’ authority in the household than girls [19]. Contrary to that, most of early adolescents in Shanghai refused stereotypical gender roles [20].

The last items addressed in the gender norms scales were about negative social repercussions for challenging gender roles, where 51% of participated students agreed that it was okay to tease a boy who acted like a girl, and 27.5% agreed that it was okay to tease a girl who acted like a boy. In contrast, only 18% of adolescents in Indonesia approved of teasing behaviors against adolescents with atypical gendered behavior [19]. This suggests that adolescents with atypical gendered behavior might be more prone to peer violence as it was reported in some studies that adolescents non-conformant to the stereotypical gender norms are often excluded and bullied [24, 25]. However, by investigating the association between gender norms and bullying behavior, our study results showed that the students’ perception of the studied gender norms does not affect the students’ status of bullying and victimization.

Lastly, the study findings regarding the association between the perception of gender norms and some sociodemographic characteristics showed that the students’ endorsement of a sexual double standard, stereotypical gender traits, and stereotypical gender roles decreases with higher education of the mothers and when the mothers are employed. The positive effect of the mother’s education and having a working mother on the gender norms of both boys and girls had been reported in many studies [26, 27]. In Shanghai, China, girls’ endorsement for stereotypical gender roles was negatively associated with their mothers’ employment status [26]. Also, in the USA, Bertrand’s study found that adolescents’ gender attitudes appear positively influenced by having a working mother [27].

### Study limitations

First, this study is cross-sectional, and thus inferences about causality are not warranted. Second, the participants were from governmental schools only, so the students in private schools were not represented in the study. Third, this study relied on a single informant assessment for bullying and victimization status, that is, relying on students’ perspectives without incorporating inputs from teachers or parents. This approach may not have yielded a comprehensive understanding of the problem.

## Conclusion

Early in adolescence, boys and girls in this study start to perceive a number of unequal gender norms related to gender relations, stereotypical norms and traits. But boys are more likely to endorse stereotypical gender traits and roles than girls and to accept heteronormative relations among adolescents, while girls are more likely to perceive a sexual double standard regarding boy/girl relationships. Moreover, boys are more likely to sanction atypical gender behaviors than girls, but both boys’ and girls’ perception of gender norms is not related to their status of bullying and victimization.

## Data Availability

The data that support the findings of this study are available from the corresponding author upon request.

## Abbreviations

SDGs: Sustainable Development Goals
GEAS: Global Early Adolescent Study
GEM scale: Gender-Equitable Men scale
